# Dissolution and ionization of sodium superoxide in sodium–oxygen batteries

**DOI:** 10.1038/ncomms10670

**Published:** 2016-02-19

**Authors:** Jinsoo Kim, Hyeokjun Park, Byungju Lee, Won Mo Seong, Hee-Dae Lim, Youngjoon Bae, Haegyeom Kim, Won Keun Kim, Kyoung Han Ryu, Kisuk Kang

**Affiliations:** 1 Department of Materials Science and Engineering, Research Institute of Advanced Materials, Seoul National University, 1 Gwanak-ro, Gwanak-gu, Seoul 151-742, Republic of Korea; 2 Center for Nanoparticle Research at Institute for Basic Science, Seoul National University, 1 Gwanak-ro, Gwanak-gu, Seoul 151-742, Republic of Korea; 3 Environment and Energy Research Team, Division of Automotive Research and Development, Hyundai Motor Company, 37 Cheoldobangmulgwan-ro, Uiwang, Gyeonggi-do 437-815, Republic of Korea

## Abstract

With the demand for high-energy-storage devices, the rechargeable metal–oxygen battery has attracted attention recently. Sodium–oxygen batteries have been regarded as the most promising candidates because of their lower-charge overpotential compared with that of lithium–oxygen system. However, conflicting observations with different discharge products have inhibited the understanding of precise reactions in the battery. Here we demonstrate that the competition between the electrochemical and chemical reactions in sodium–oxygen batteries leads to the dissolution and ionization of sodium superoxide, liberating superoxide anion and triggering the formation of sodium peroxide dihydrate (Na_2_O_2_·2H_2_O). On the formation of Na_2_O_2_·2H_2_O, the charge overpotential of sodium–oxygen cells significantly increases. This verification addresses the origin of conflicting discharge products and overpotentials observed in sodium–oxygen systems. Our proposed model provides guidelines to help direct the reactions in sodium–oxygen batteries to achieve high efficiency and rechargeability.

## Abstract (web summary)

Sodium-oxygen batteries are promising energy storage devices but the nature of their discharge products remains unresolved. Here, the authors reveal that the dissolution and ionization of sodium superoxide leads to the formation of other phases, which increases the charge overpotential of the cell.

## Graphical abstract

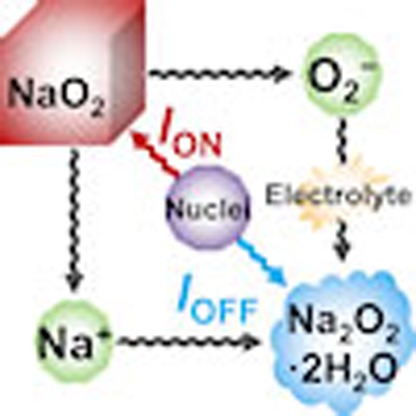

To address the increasing use of renewable energy and launch of electric vehicles, the need for rechargeable batteries with high-energy densities has been growing more rapidly than ever before^1^,^2^. Among the available battery chemistries, metal–oxygen systems offer the highest energy density with the largest theoretical capacities. Unlike conventional lithium-ion batteries, the direct reaction between oxygen and light metals such as lithium and sodium in metal–oxygen systems circumvents the need for a heavy transition metal redox couple in their operation, thereby making a high gravimetric energy density achievable^3^,^4^,^5^,^6^,^7^. The most intensively studied metal–oxygen system to date is the lithium–oxygen (Li–O_2_) battery, which shares a similar lithium chemistry with lithium-ion batteries. However, this system suffers from poor cycle stability and efficiency, which has retarded the feasibility of its use in practical systems^8^,^9^. In particular, the large charge overpotential over 1 V, the main reason for the low efficiency, also accelerates the degradation of the electrode and electrolyte^9^. As an alternative, Na has been introduced to replace Li in Li–O_2_ batteries with a few important merits^10^. Despite the reduction in the energy density resulting from the lower redox potential of Na/Na^+^, Na resources are readily available, are less expensive than Li and can easily replace Li in the battery chemistry. It has been reported that the redox reactions in the sodium–oxygen (Na–O_2_) battery result in an extremely low charge overpotential (∼0.2 V) despite involving the formation of micrometre-sized sodium superoxide (NaO_2_) cubic crystallites^11^,^12^,^13^,^14^. This unique phenomenon supports the idea that this system is a promising alternative not only in terms of the cost of materials but also regarding the potential practical performance advantages.

Notably, however, the reactions of Na–O_2_ cells appear to be more diverse than those of Li–O_2_ cells. Contrary to the initial report of NaO_2_ discharge products, some recent lines of work could not reproduce either the formation of the discharge product NaO_2_ or the low charge overpotential^15^,^16^,^17^. It was reported that sodium peroxide (Na_2_O_2_)^15^,^16^,^17^ or sodium peroxide dihydrate (Na_2_O_2_·2H_2_O)^18^,^19^ was formed instead. In addition, these cells exhibited high overpotential during charge, similar to that observed in the Li–O_2_ system. Many groups have attempted to determine the reasons for these discrepancies; however, to date, the main cause of the divergence of reactions has not been identified. Janek *et al*. investigated the effect of different carbon electrodes; Guo *et al*. and Shao-Horn *et al*. addressed this issue but observed no critical differences among the cases^12^,^20^,^21^.

In this work, we demonstrate the interplay of the diverse reactions in Na–O_2_ batteries involving a series of electrochemical and chemical reactions as a function of time. Under systematic control of the operating conditions, we observe that the galvanostatic charge/discharge profiles are sensitively affected by the conditions and durations of the electrochemical operations. It is also revealed that the electrochemically formed NaO_2_ is unstable and degrades into Na_2_O_2_·2H_2_O in the absence of an applied current. The spontaneous dissolution and ionization of NaO_2_ liberates the free O_2_^−^ in the electrolyte and promotes side reactions involving the formation of Na_2_O_2_·2H_2_O. On the basis of these observations, we propose reaction mechanisms of Na–O_2_ batteries under various operating conditions. This report is the first to reveal the relationships among the different discharge products observed in Na–O_2_ batteries, which broadens our understanding of the electrochemical and chemical reactions in Na–O_2_ batteries. Furthermore, these discussions may offer insight and guidance to the metal–air battery community in terms of regulating the kinetics of the intertwined reactions.

## Results

### Electrochemical profile depending on the operating condition

**Figure 1:**
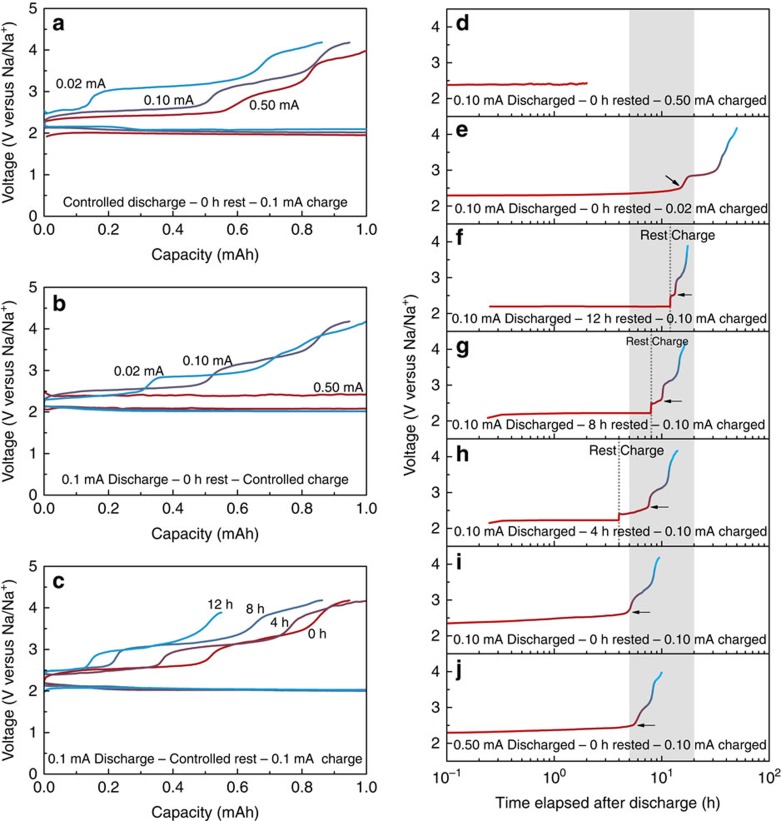
Electrochemical charge/discharge profiles of Na–O_2_ cells under various operating conditions. (**a**) Discharge currents of 0.02, 0.1 and 0.5 mA; (**b**) charge currents of 0.02, 0.1 and 0.5 mA and (**c**) rest times of 0, 4, 8 and 12 h. All the cells utilized a limited capacity of 1.0 mAh. (**d**–**j**) Representations of voltage profiles as a function of time corresponding to **a**–**c**. The shaded area indicates that range of the first points of the polarized charge potentials.

To address the previous conflicting results on the discharge products and overpotentials of Na–O_2_ cells, we carefully assessed the effects of operating parameters on the resulting electrochemical profiles. Among the various parameters examined (Supplementary Figs 1–3 and Supplementary Notes 1–3), we observed that the charge/discharge profiles were most sensitively affected by the applied current and rest time between the discharge and charge, which was analogous to the report by Yadegari *et al*. as a function of discharge current or limited capacities^19^. Figure 1 presents and compares the electrochemical profiles obtained under various conditions. Although the discharge profiles are similar, with a single plateau at ∼2.1 V, there are roughly three different charging plateaus observed at (i) ∼2.5 V, (ii) ∼3.0 V and (iii) 3.8 V, which agree with recent reports under certain settings^19^,^20^. However, the relative lengths of each plateau markedly vary under differing operating conditions. For the cases of controlled discharge currents followed by a constant current charging in Fig. 1a, it was observed that the length of the lower plateau (∼2.5 V) in the charge profiles was reduced as the applied discharge current decreased from 0.5 to 0.02 mA. However, the lengths of the plateaus at higher voltages, that is, ∼3.0 and 3.8 V, were substantially increased, resulting in an overall larger overpotential. Similar behaviours were observed in Fig. 1b when varying the charge currents after a constant current discharge. With the lower applied charge currents, the cell exhibited a higher charging overpotential with shortened plateau length at 2.5 V. This result contrasts with the general observation that slow charging/discharging of electrochemical cells results in a voltage close to the equilibrium potential, thereby resulting in smaller overpotentials. In addition, this result strongly indicates that the different discharge products might undergo the charging process at each case. Notably, the shapes of the electrochemical charge profiles provide important clues to determine the discharge products of Na–O_2_ reactions^19^,^22^. Even though the discharge products should be identical for the cases of the same protocol of discharge, each charge profile was distinct with different charge currents. This finding implies that the initial discharge products are gradually transformed into other phases during the charge process via time-dependent reactions. To verify whether this transformation occurs via an electrochemical or chemical reaction, we also controlled the rest time between the discharge and charge processes. As observed in Fig. 1c, the lowest voltage region in the charge profiles systematically decreases on increasing the rest time from 0 to 12 h. The change in the electrochemical profile in the absence of the applied current clearly indicates that the time-dependent chemical reactions occurred during the rest period, affecting the subsequent charging. This behaviour was also confirmed in similar tests for the higher charging currents with the resting time after the discharge, which revealed the growth of the charge polarizations on increasing the rest time (Supplementary Fig. 1).

The time-dependent chemical reactions can be more clearly visualized by plotting all the voltage profiles as a function of the time. Figure 1d–j illustrates the voltage evolution of each cell after the completion of the discharge at different operating conditions. The first inflection points of the voltage profiles at charge (indicated with arrows) occur at ∼10 h regardless of the rest or charge protocols. This result indicates that a specific time of ∼10 h is required before observing a change of the profile, which hints at the kinetics of the chemical reactions.

### Time-resolved characterization of discharge products

**Figure 2:**
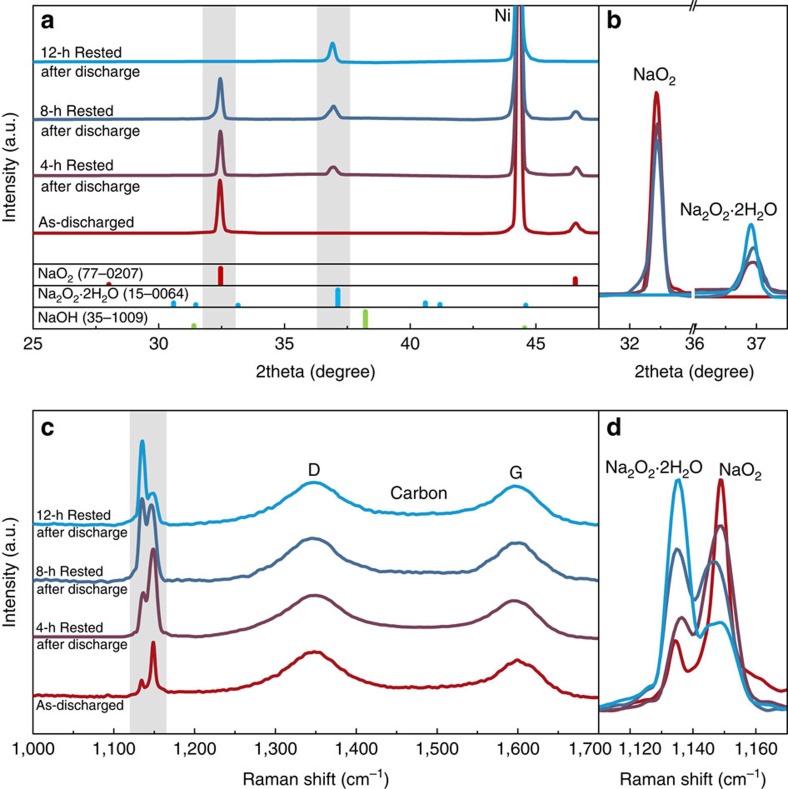
Time-resolved characterization showing the phase transitions of the discharge products of the Na–O_2_ cells. (**a**,**b**) X-ray diffraction spectra of the discharged cathodes of Na–O_2_ batteries with rest times of 0, 4, 8 and 12 h. (**c**,**d**) Raman spectra of the discharged cathodes of Na–O_2_ batteries with rest times of 0, 4, 8 and 12 h.

To confirm the time-dependent phase transformation of the discharge products via chemical reaction in Na–O_2_ cells, we characterized the discharge products in air electrodes as a function of the rest time. The highly crystalline NaO_2_ was observed directly after the discharge with no other phases, as demonstrated in the X-ray diffraction spectra (Fig. 2a,b)^11^. However, after being aged for several hours, the NaO_2_ peak slowly diminished, whereas the characteristic peak of Na_2_O_2_·2H_2_O began to appear and grew. After 12 h of resting, the initial discharge product was completely transformed into Na_2_O_2_·2H_2_O. It should be noted that Na_2_O_2_·2H_2_O has often been regarded as a main discharge product in previous reports of Na–O_2_ batteries^18^,^19^,^20^. Recently, Ortiz-Vitoriano *et al*. reported that NaO_2_ could convert to Na_2_O_2_·2H_2_O on exposure to the ambient air during the characterization at room temperature^21^. However, our data show that such transformation occurs in the electrochemical cells by the intrinsic dissolving characteristics of NaO_2_ in the electrolyte even without the exposure to the ambient atmosphere. Remarkably, the time taken for the discharge product to completely transform into Na_2_O_2_·2H_2_O coincides with the timeline of Fig. 1d–j, which shows the inflection of the voltage rising after ∼10 h. When we analysed the phases of the discharge products as a function of the applied discharge currents (Supplementary Fig. 4), it was also observed that the NaO_2_/Na_2_O_2_·2H_2_O ratio decreased with the lower operating current, which is consistent with the time-dependent transformation of the discharge products.

Raman spectroscopy results confirmed that the initial NaO_2_ discharge products gradually transformed into Na_2_O_2_·2H_2_O with resting. In Fig. 2c, the two distinct peaks of NaO_2_ and Na_2_O_2_·2H_2_O are detected along with the characteristic bands (D/G) of the carbon electrode. The Raman signals at 1,156 and 1,136 cm^−1^ are attributed to the O–O stretch bonding in NaO_2_ and Na_2_O_2_·2H_2_O, respectively^21^. The systematic change in the relative ratios of NaO_2_ and Na_2_O_2_·2H_2_O with time is clearly illustrated in Fig. 2d, which agrees well with the results in Fig. 2b. The phase transition of NaO_2_ to proton-containing Na_2_O_2_·2H_2_O indicates a source of protons in the electrochemical cell. Considering the low water content in the electrolyte used for the cell (less than ∼5 p.p.m.), which is insufficient to form the phase (calculations provided in Supplementary Note 4)^21^,^23^,^24^, the protons are likely delivered from other sources such as the electrolyte solvent. As we could expect, the rechargeability of Na–O_2_ cell was better for the highly biased electrochemical conditions coupled with the low polarized charge profile (Supplementary Fig. 3), which is attributed to the electrochemical formation and decomposition of NaO_2_ as shown in Supplementary Fig. 5. However, the electrochemical reversibility with the three-stepped charge profile shown from Na_2_O_2_·2H_2_O was relatively worse compared with the former conditions. The proposed transformation mechanism will be discussed in detail later.

### Morphological change of discharge products over time

**Figure 3:**
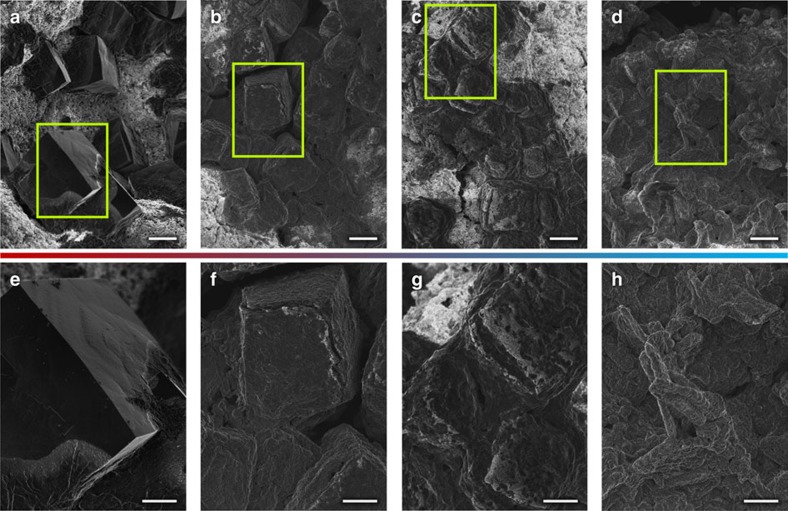
Time-resolved examinations of the morphology of discharge products on the cathodes of Na–O_2_ cells. (**a**–**d**) Morphology of the discharge products of Na–O_2_ cells (scale bar, 10 μm). (**e**–**h**) Corresponding magnified scanning electron microscopy micrographs (scale bar, 5 μm); (**a**,**e**) as-discharged, (**b**,**f**) 4-h rest after discharge, (**c**,**g**) 8-h rest after discharge and (**d**,**h**) 12-h rest after discharge.

To visualize the transition process, we examined the morphologies of the discharge products at different rest times within 12 h. In Fig. 3a, well-defined micron-sized cubic NaO_2_ was observed immediately after the discharge, which agrees with the observation of Hartmann *et al*.^11^. However, the edges of the cubes became significantly dull, and the overall shapes of the cubes obtained were smudged during the rest period (Fig. 3b,c). At the end of the rest period, the cubic crystallites completely disappeared, and rod-shaped microparticles began to appear, which resemble the Na_2_O_2_·2H_2_O in a previous report^19^. This morphological change suggests the disappearance of NaO_2_ and the subsequent appearance of Na_2_O_2_·2H_2_O in the cell during the rest period. Moreover, this finding implies that the transformation does not occur via a conventional solid-state or interfacial reaction between NaO_2_ and the electrolyte to form Na_2_O_2_·2H_2_O, which would not involve significant morphological change. Rather, it is likely to be a solution-mediated process through dissolution and nucleation^25^,^26^,^27^.

### Dissolution and ionization of NaO_2_

**Figure 4:**
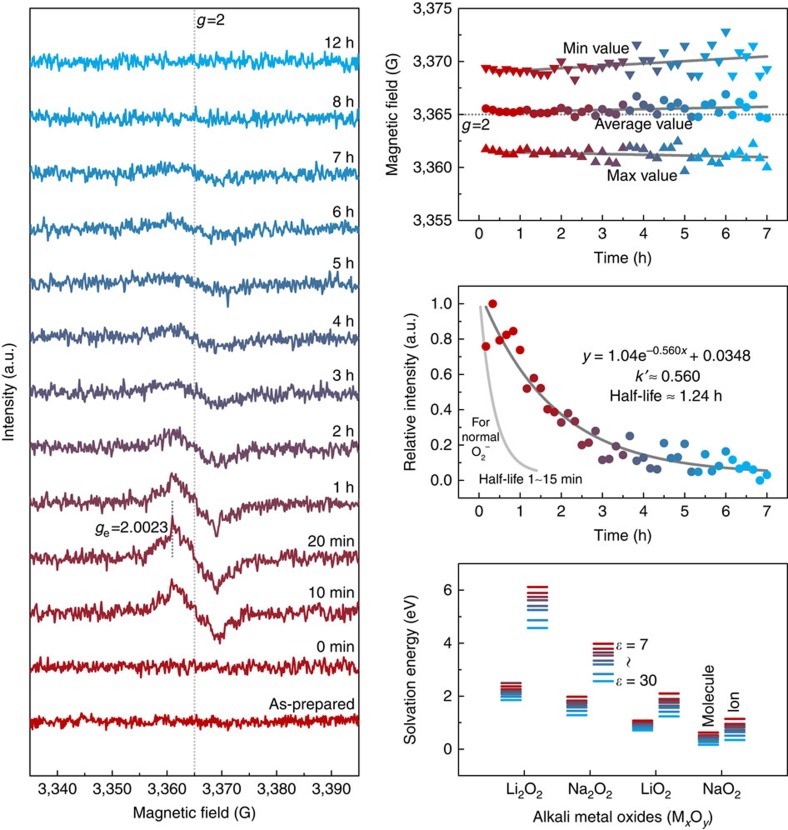
ESR analysis and theoretical calculations of the dissolution and ionization of NaO_2_ into the electrolyte. (**a**) Time-dependent ESR measurements for the fresh electrolytes (0.5 M NaCF_3_SO_3_ in diethylene glycol dimethyl ether) with soaking of the pre-discharged cathode without any aging. (**b**) Maximum, minimum and average values of ESR signals as a function of time. (**c**) Exponential decay of ESR signals and the common trend line of O_2_^−^. (**d**) Calculations of the solvation energy for several alkali-metal superoxides and peroxides with the various dielectric constants (*ɛ*=7∼30).

We investigated the possibility of the dissolution of the solid NaO_2_ phase in the electrolyte using electron spin resonance (ESR) spectroscopy, which is useful for detecting the magnetic responses of the unpaired electrons in radicals such as O_2_^−^ (ref. 28). Surprisingly, as observed in Fig. 4a, with the simple immersion of the pre-discharged cathodes, the ESR signal evolved within 10 min from the fresh electrolyte, indicating the presence of O_2_^−^. To avoid any effect of the remaining oxygen from the disassembled Na–O_2_ cells, the pre-discharged cathodes were washed with fresh electrolyte before the measurement, which led to an identical result. The calculated *g*-value of 2.0023 for the observed ESR signal corresponds well with the theoretical value of the unpaired electron in free O_2_^−^ (ref. 29). The solubility of NaO_2_ in the electrolyte was roughly estimated to be ∼187 mM, which is in the similar order with the report by Schechter *et al*.^30^, but has a relatively large discrepancy to the report by Hartmann *et al*.^31^. This discrepancy might be mainly due to the additional chemical reactions involving the precipitation of solid Na_2_O_2_·2H_2_O. The detection of O_2_^−^ indicates that the NaO_2_ is soluble in the ether-based electrolytes, which was also expected from the literatures with the electrochemical determinations^21^,^24^,^31^. Furthermore, this behaviour is analogous to highly soluble LiO_2_ in the solvatable conditions of Li–O_2_ batteries^23^,^32^. More importantly, the dissolution can immediately lead to the ionization of NaO_2_, liberating O_2_^−^, the consequences of which will be discussed later.

Figure 4b shows that the peak widths of the ESR signals increased slightly with time. The broadening indicates the energy exchange of the spin with the local environments via spin–spin relaxation or spin–lattice relaxation^28^. This interaction supports the time-dependent chemical reactions associated with the dissolved O_2_^−^ with its neighbouring electrolyte solvent. The intensity of the O_2_^−^ signal is the highest ∼20 min after the immersion and exponentially decreases over time, indicating the instability of O_2_^−^ in the electrolyte^33^. From this behaviour, we could derive that it was a pseudo-first-order reaction that mainly relates with the concentration of O_2_^−^. On the basis of the exponential fitting of the relative intensity of ESR signals, the pseudo-first-order rate constant of H^+^ abstraction was obtained as ∼*k*′≈0.560, and its corresponding half-life was estimated as ∼*t*_1/2_=ln (2)/*k*′≈1.24 h (the detailed derivation is in Supplementary Note 5). Figure 4c reveals, however, that the time-dependent decay of the intensity is relatively sluggish compared with the intrinsic lifetime of normal O_2_^−^. Typically, the half-life of O_2_^−^ is ∼1–15 min because of its high reactivity and instability^34^. The abnormally long half-life in the electrolyte (∼1.24 h) in our case is believed to occur because O_2_^−^ is continuously generated with the dissolution of NaO_2_. The ESR signal completely vanished after ∼8 h, which is slightly faster than the time required for the formation of Na_2_O_2_·2H_2_O in Fig. 2. Despite the evolution of O_2_^−^, the overall signal decay might be induced from the relatively dominant H^+^ abstraction due to the reactivity of O_2_^−^. This gap in the kinetics might originate from the time required to form the Na_2_O_2_·2H_2_O phase from the O_2_^−^.

To understand the dissolution and ionization behaviour of NaO_2_, the solvation energies of various alkali-metal superoxides and peroxides were calculated for comparison using first-principle calculations with the series of dielectric constant from 7 to 30. Figure 4d reveals that generally the superoxide exhibits a lower solvation energy than the peroxide for both lithium and sodium compounds. This result is consistent with our result of NaO_2_ dissolution and the recent experimental findings for Li–O_2_ batteries, which indicated that LiO_2_ is found mostly as soluble intermediates in the electrolyte in contrast to the solid phase of Li_2_O_2_ (refs 23, 32). In addition, it is notable that the solvation energy of the sodium phases was significantly lower than that of the lithium phases, which is attributed to the weaker Lewis acidity of the Na cation compared with that of the Li cation in the polar solvent^35^,^36^. However, for the solvents with substantially lower dielectric constant (*ɛ*=∼7), the dissolution is not favourable even in NaO_2_. Molecular dissolution energies of NaO_2_ in model solvents are ∼0.6 eV, which roughly corresponds to one molecule dissolution among 10^10^ formula units of NaO_2_. On the other hand, it markedly diminishes to 0.17 eV (one molecule among 10^3^ formula units of NaO_2_) in *ɛ*=30 (ref. 37). Note that, for the low dielectric constant solvents, dielectric constant of the solution sensitively increases with increasing salt concentration, which can result in higher solution dielectric constant than that of the pure solvent^38^. Therefore, it is expected that the dissolution of NaO_2_ can occur when salts are present in the electrolyte, which is consistent with the observation of O_2_^−^ in the ESR analysis. It also is noted that even with the dissolving characteristics of NaO_2_, the crystallization of NaO_2_ is possible in the normal discharging conditions with the supersaturation of localized reactants such as Na^+^ and O_2_^−^ (refs 21, 31, 39, 40). In the other case where the supply of the reactants such as Na^+^ is limited, for example, in the absence of the applied voltage, the dissolution and ionization might dominate, giving rise to the formation of Na_2_O_2_·2H_2_O as a discharge product.

### Proposed mechanism of Na–O_2_ batteries

**Figure 5:**
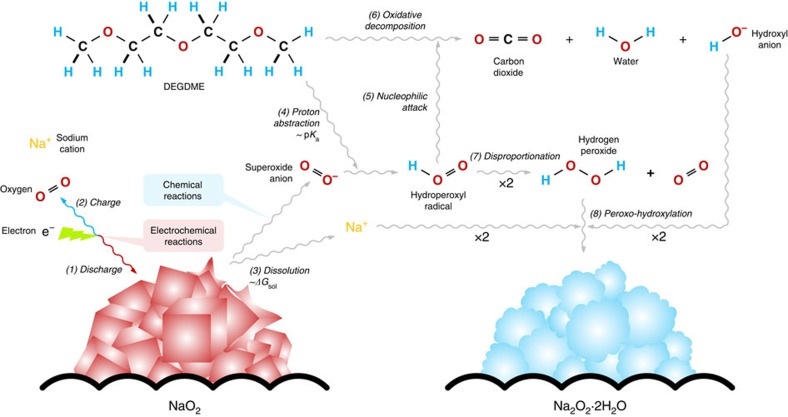
Schematic of the proposed mechanism illustrating the electrochemical and chemical reactions under various operating conditions. For the electrochemical reaction, NaO_2_ is formed and decomposed during discharge/charge (Reactions 1 and 2). For the chemical reaction, NaO_2_ is dissolved and ionized into the electrolyte (Reaction 3), which promotes the undesired degradation of the electrolyte (Reactions 4–6). Na_2_O_2_·2H_2_O is formed during the subsequent chemical reactions (Reactions 7 and 8).

On the basis of the previous reports and our new findings, we propose a mechanism that describes the electrochemical and chemical reactions in Na–O_2_ systems in Fig. 5. The well-established discharge process^11^ can be illustrated with the reduction of an O_2_ molecule into O_2_^−^, which reacts with Na^+^ to form NaO_2_ (Reaction 1), and the charge process is the reverse reaction (Reaction 2). After or during the discharge, the NaO_2_ is prone to dissolution and ionization into the electrolyte based on the solvating energy (*ΔG*_sol_) in the solvent (Reaction 3)^32^. The dissolution of NaO_2_ generates O_2_^−^, which can degrade the surrounding molecules because of its chemical instability. Typically, the liberated O_2_^−^ is a strong reagent for the abstraction of H^+^ from the electrolyte solvents (Reaction 4)^9^, and the degree of H^+^ abstraction^41^,^42^ is determined by the acid-dissociation constant (p*K*_a_) of the solvent. Some hydroperoxyl radicals (HO_2_) might be formed during this process, resulting in the nucleophilic attack of the H^+^-lost solvent (Reaction 5)^43^. However, the evolution of HO_2_ can be helpful in promoting the solution-mediated discharge/charge process as recently reported by Xia *et al*.^24^. Nevertheless, in a circumstance where the dissolution/ionization of NaO_2_ is dominant, the liberation of O_2_^−^ is overwhelmingly larger than a possible HO_2_ formation, inducing the H^+^ abstraction from the neighbouring electrolyte solvent. Meanwhile, the solvent undergoes oxidative decompositions to produce byproducts such as carbon dioxide (CO_2_), water (H_2_O) and hydroxyl anions (OH^−^; Reaction 6)^9^. It is also possible that the coupling of HO_2_ leads to disproportionation into hydrogen peroxide (H_2_O_2_) and O_2_ (Reaction 7)^44^. In the presence of both Na^+^ and OH^−^, which is effectively the dissolution state of sodium hydroxide (NaOH), a solid crystallite of NaOH can precipitate with a higher concentration of OH^−^ produced. Further reaction between NaOH and H_2_O_2_ from Reaction 7 leads to the formation of Na_2_O_2_·2H_2_O via peroxo-hydroxylation, whose reverse reaction is well known (Reaction 8)^45^. To support our proposed reaction mechanism, we chose several intermediate reactions that should be verified according to the reaction model in the Supplementary Information. Supplementary Figs 6–9 demonstrate that O_2_^−^ plays an important role after the dissolution of NaO_2_ in converting the discharge product to Na_2_O_2_·2H_2_O via degradation of the electrolyte involving OH^−^ and H_2_O_2_. These identifications strongly support the proposed mechanism of competing electrochemical and following chemical reactions in Na–O_2_ batteries. The detailed discussions are provided in Supplementary Notes 6–9. The reaction equations are summarized below:


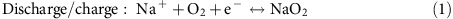



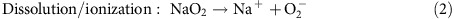



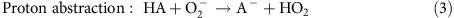



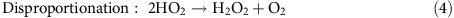



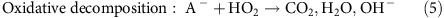



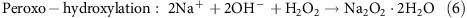


It is noteworthy that a similar behaviour has been recently reported for reactions in Li–O_2_ batteries. The solvating environment was demonstrated to alter the stability of the intermediates, such as a lithium superoxide (LiO_2_), thus affecting the overall reaction paths^23^,^32^. LiO_2_ is a precedent phase with the direct reaction of a Li cation and superoxide anion (O_2_^−^), which readily decomposes into lithium peroxide (Li_2_O_2_) via either an electrochemical surface reaction or disproportionation^23^,^32^. Although LiO_2_ is known to be unstable^46^,^47^, it was recently demonstrated that LiO_2_ might be dissolved into the electrolyte and aid in the formation of the toroidal Li_2_O_2_ via a solution reaction under highly solvating conditions^23^. NaO_2_ shares this dissolving nature with LiO_2_ even though the thermodynamic stability of NaO_2_ warrants its formation as a discharge product. The significant dissolution of NaO_2_ supports the conclusion that the dominant reaction in Na–O_2_ batteries relies on the solution-mediated reactions of nucleation and growth of NaO_2_ (refs 21, 24, 31) and implies that the capacities and morphology of the reaction products would be greatly affected by the energetics of NaO_2_ under various conditions (such as different electrolytes and current rates). This is also supplemented with the recently reported observations^21^,^31^ and operating mechanism^24^ in terms of the various states of electrochemical and chemical reactions.

## Discussion

We successfully demonstrated the interplay of the diverse competing reactions in Na–O_2_ batteries. The time-dependent chemical reactions were identified as being triggered from the dissolution and ionization of the electrochemically formed NaO_2_ in the electrolyte. The liberated O_2_^−^ reacts with the electrolyte solvent to form Na_2_O_2_·2H_2_O following a series of intermediate steps. The Na_2_O_2_·2H_2_O in the air electrode requires a higher energy for the decomposition, which leads to the increased charge overpotential and irreversibility of Na–O_2_ cells. This report is the first to correlate the electrochemical and chemical reactions with the operating conditions in Na–O_2_ batteries, and our findings concerning the relationships among different phases resolve the conflicting observations of different discharge products in previous Na–O_2_ batteries. To prepare a better performing Na–O_2_ battery, a strategy to prevent the transformation of NaO_2_ into Na_2_O_2_·2H_2_O while still allowing the solution-mediate discharge reaction is necessary. We hope that the findings of this study can provide a basis for researchers to navigate and direct the reactions in Na–O_2_ batteries to achieve high efficiency and rechargeability.

## Methods

### Cell assembly and galvanostatic cycling of Na–O_2_ cells

The carbon cathode was prepared by casting Ketjen Black carbon paste and polytetrafluoroethylene (60 wt% emersion in water, Sigma-Aldrich) with a mass ratio of 9:1 in a solution of isopropanol (>99.7%, Sigma-Aldrich) and N-methyl-2-pyrrolidone (99.5%, anhydrous, Sigma-Aldrich) with a volume ratio of 1:1 on Ni mesh current collectors. The carbon-coated Ni mesh was dried at 120 °C and heated at 400 °C for 4 h in Ar to completely remove any residual H_2_O impurities.

All the procedures described below were performed in an Ar-filled glove box (O_2_ level<0.5 p.p.m. and H_2_O level<0.5 p.p.m.). The Na–O_2_ cells were assembled as a Swagelok-type cell with stacking of the Na metal anode, electrolyte-soaked separators and carbon cathode, which was punched with a half-inch diameter. The Na metal anode was carefully prepared by milling dry Na metal chunks (ACS Reagent, Sigma-Aldrich) after removing the contaminated surfaces. The electrolyte was prepared with diethylene glycol dimethyl ether (anhydrous, 99.5%, Sigma-Aldrich), which contains 0.5 M NaCF_3_SO_3_ (98%, Sigma-Aldrich). The solvent was dried using 3-Å molecular sieves for over 1 week, and the salt was also kept in a vacuum oven at 180 °C for the same time before use. The final water content in the electrolyte was less than 10 p.p.m. according to a Karl–Fisher titration measurement. The amount of electrolyte used for the cell was 200 μl. Two sheets of Celgard 2400 were used as separators. Electrochemical battery tests of the Na–O_2_ cells were conducted using a potentio-galvanostat (WonA Tech, WBCS 3000, Korea). All the cells were relaxed under 770 torr of O_2_ pressure for 10 min before the tests. After being saturated with O_2_ gas, the cells were operated in the closed state with a limited capacity of 1 mAh, lower voltage cutoff of 1.6 V and upper voltage cutoff of 4.2 V. Special protocol based on a pulsed current was applied during the charge to avoid dendritic failure of the Na metal anode. The on/off time ratio of the pulse charge was 1:4 (applying current for 0.5 s and relaxing for 2 s). More details about our charge protocol are provided in Supplementary Figs 10 and 11 and following discussions in Supplementary Note 10.

### Characterization of Na–O_2_ cells

The discharged cathodes after the different rest times were collected from disassembled Na–O_2_ cells and washed with acetonitrile (anhydrous, 99.8%, Sigma-Aldrich) in a glove box to remove any residual electrolyte. X-ray diffraction spectra of the cathodes were obtained using a Bruker D2-Phaser (Cu Kα *λ*=1.5406 Å) with the aid of a specially designed air-tight holder to prevent outer atmospheric contamination. Raman spectra were obtained using a Horiba Jobin-Yvon LabRam Aramis spectrometer (the 514-nm line of an Ar–ion laser was used as the excitation source). The scattered light of the Raman signal was collected in a backscattering geometry using the × 50 microscope objective lens. Field-emission scanning electron microscopy (MERLIN Compact, ZEISS, Germany) was used for the morphological observations after Pt coating. For ESR characterization, the collected powder from the discharged cathodes after rinsing to remove the residual used electrolytes was soaked in fresh electrolyte. After immersing the powdery discharged cathodes, the ESR signal of the electrolytes was measured at room temperature using a JEOL JES-TE200 ESR spectrometer every 10 min for 12 h using a liquid quartz cell. The microwave X-band frequency was 9.42 GHz at 1-mW power.

### Theoretical calculations of solvation energy

First-principles calculations were performed using the spin-polarized generalized gradient approximation. A continuum solvation model (VASPsol^48^,^49^ code) was used to evaluate the solvation energy of the alkali-metal superoxide/peroxide (M_*x*_O_2_, M: Li, Na, *x*=1 or 2). The following equations were used considering both the (1) molecular and (2) ionized solvated states:


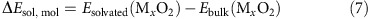



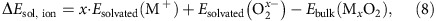


where *E*_solvated_(M_*x*_O_2_) and *E*_bulk_(M_*x*_O_2_) are the total energies of the solvated and bulk M_*x*_O_2_ per formula unit, respectively. The solvated species (ions or molecules) were placed in a 13 Å × 13 Å × 13 Å cell as an isolated species. We used the plane-wave basis with an energy cutoff of 550 eV and a Monkhorst-Pack 2 × 2 × 2 *k*-point mesh. On the basis of previous reports^50^,^51^ that stated that the solvation entropy term (TS) of polar molecules and ions in the standard state is less than 5% of the enthalpy term (*H*), we neglected the entropy effect of the solvation in these calculations.

## Additional information

**How to cite this article:** Kim, J. *et al*. Dissolution and ionization of sodium superoxide in sodium–oxygen batteries. *Nat. Commun.* 7:10670 doi: 10.1038/ncomms10670 (2016).

## Supplementary Material

Supplementary Information Supplementary Figures 1-11, Supplementary Notes 1-10 and Supplementary References

## References

[b1] Tarascon J. M. & Armand M. Issues and challenges facing rechargeable lithium batteries. Nature 414, 359–367 (2001). 11713543 10.1038/35104644

[b2] Kang K., Meng Y. S., Bréger J., Grey C. P. & Ceder G. Electrodes with high power and high capacity for rechargeable lithium batteries. Science 311, 977–980 (2006). 16484487 10.1126/science.1122152

[b3] Abraham K. M. & Jiang Z. A polymer electrolyte-based rechargeable lithium/oxygen battery. J. Electrochem. Soc. 143, 1–5 (1996).

[b4] Bruce P. G., Freunberger S. A., Hardwick L. J. & Tarascon J.-M. Li-O_2_ and Li-S batteries with high energy storage. Nat. Mater. 11, 19–29 (2012). 22169914 10.1038/nmat3191

[b5] Peng Z., Freunberger S. A., Chen Y. & Bruce P. G. A reversible and higher-rate Li-O_2_ battery. Science 337, 563–566 (2012). 22821984 10.1126/science.1223985

[b6] Ottakam Thotiyl M. M. . A stable cathode for the aprotic Li-O_2_ battery. Nat. Mater. 12, 1050–1056 (2013). 23995325 10.1038/nmat3737

[b7] Lim H.-D. . Superior rechargeability and efficiency of lithium-oxygen batteries: hierarchical air electrode architecture combined with a soluble catalyst. Angew. Chem. Int. Ed. 126, 4007–4012 (2014). 10.1002/anie.201400711 24596170

[b8] Débart A., Paterson A. J., Bao J. & Bruce P. G. α-MnO_2_ nanowires: a catalyst for the O_2_ electrode in rechargeable lithium batteries. Angew. Chem. Int. Ed. 47, 4521–4524 (2008). 10.1002/anie.200705648 18461594

[b9] Freunberger S. A. . The lithium-oxygen battery with ether-based electrolytes. Angew. Chem. Int. Ed. 50, 8609–8613 (2011). 10.1002/anie.201102357 21805544

[b10] Yabuuchi N., Kubota K., Dahbi M. & Komaba S. Research development on sodium-ion batteries. Chem. Rev. 114, 11636–11682 (2014). 25390643 10.1021/cr500192f

[b11] Hartmann P. . A rechargeable room-temperature sodium superoxide (NaO_2_) battery. Nat. Mater. 12, 228–232 (2013). 23202372 10.1038/nmat3486

[b12] Bender C. L., Hartmann P., Vračar M., Adelhelm P. & Janek J. On the thermodynamics, the role of the carbon cathode, and the cycle life of the sodium superoxide (NaO_2_) battery. Adv. Energy Mater. 4, 1301863 (2014).

[b13] McCloskey B. D., Garcia J. M. & Luntz A. C. Chemical and electrochemical differences in nonaqueous Li-O_2_ and Na-O_2_ batteries. J. Phys. Chem. Lett. 5, 1230–1235 (2014). 26274476 10.1021/jz500494s

[b14] Hartmann P. . Pressure dynamics in metal-oxygen (metal-air) batteries: a case study on sodium superoxide cells. J. Phys. Chem. C 118, 1461–1471 (2014).

[b15] Liu W., Sun Q., Yang Y., Xie J.-Y. & Fu Z.-W. An enhanced electrochemical performance of a sodium-air battery with graphene nanosheets as air electrode catalysts. Chem. Commun. 49, 1951–1953 (2013). 10.1039/c3cc00085k 23370447

[b16] Li Y. . Superior catalytic activity of nitrogen-doped graphene cathodes for high energy capacity sodium-air batteries. Chem. Commun. 49, 11731–11733 (2013). 10.1039/c3cc46606j 24136098

[b17] Hu Y. . Porous perovskite calcium-manganese oxide microspheres as an efficient catalyst for rechargeable sodium-oxygen batteries. J. Mater. Chem. A 3, 3320–3324 (2015).

[b18] Kim J., Lim H.-D., Gwon H. & Kang K. Sodium-oxygen batteries with alkyl-carbonate and ether based electrolytes. Phys. Chem. Chem. Phys. 15, 3623–3629 (2013). 23386220 10.1039/c3cp43225d

[b19] Yadegari H. . On rechargeability and reaction kinetics of sodium-air batteries. Energy Environ. Sci. 7, 3747–3757 (2014).

[b20] Zhao N., Li C. & Guo X. Long-life Na-O_2_ batteries with high energy efficiency enabled by electrochemically splitting NaO_2_ at a low overpotential. Phys. Chem. Chem. Phys. 16, 15646–15652 (2014). 24958445 10.1039/c4cp01961j

[b21] Ortiz-Vitoriano N. . Rate-dependent nucleation and growth of NaO_2_ in Na-O_2_ batteries. J. Phys. Chem. Lett. 6, 2636–2643 (2015). 26266746 10.1021/acs.jpclett.5b00919

[b22] Yadegari H. . Three-dimensional nanostructured air electrode for sodium-oxygen batteries: a mechanism study toward the cyclability of the cell. Chem. Mater. 27, 3040–3047 (2015).

[b23] Aetukuri N. B. . Solvating additives drive solution-mediated electrochemistry and enhance toroid growth in non-aqueous Li-O_2_ batteries. Nat. Chem. 7, 50–56 (2015). 25515890 10.1038/nchem.2132

[b24] Xia C., Black R., Fernandes R., Adams B. & Nazar L. F. The critical role of phase-transfer catalysis in aprotic sodium-oxygen batteries. Nat. Chem. 7, 496–501 (2015). 25991528 10.1038/nchem.2260

[b25] Schroeder M. A. . DMSO-Li_2_O_2_ interface in the rechargeable Li-O_2_ battery cathode: theoretical and experimental perspectives on stability. ACS Appl. Mater. Interfaces 7, 11402–11411 (2015). 25945948 10.1021/acsami.5b01969

[b26] Kumar N., Radin M. D., Wood B. C., Ogitsu T. & Siegel D. J. Surface-mediated solvent decomposition in li-air batteries: impact of peroxide and superoxide surface terminations. J. Phys. Chem. C 119, 9050–9060 (2015).

[b27] Kwabi D. G. . Chemical instability of dimethyl sulfoxide in lithium-air batteries. J. Phys. Chem. Lett. 5, 2850–2856 (2014). 26278088 10.1021/jz5013824

[b28] Wang Q., Yang X.-Q. & Qu D. In situ ESR spectro-electrochemical investigation of the superoxide anion radical during the electrochemical O_2_ reduction reaction in aprotic electrolyte. Carbon 61, 336–341 (2013).

[b29] Eastland G. W. & Symons M. C. Electron spin resonance studies of superoxide ions produced by radiolysis in alcoholic media. J. Phys. Chem. 81, 1502–1504 (1977).

[b30] Schechter D. L. & Kleinberg J. Reactions of some metal salts with alkali superoxides in liquid ammonia. J. Am. Chem. Soc. 76, 3297–3300 (1954).

[b31] Hartmann P. . Discharge and charge reaction paths in sodium-oxygen batteries: does NaO_2_ form by direct electrochemical growth or by precipitation from solution? J. Phys. Chem. C 119, 22778–22786 (2015).

[b32] Johnson L. . The role of LiO_2_ solubility in O_2_ reduction in aprotic solvents and its consequences for Li-O_2_ batteries. Nat. Chem. 6, 1091–1099 (2014). 25411888 10.1038/nchem.2101

[b33] Hall P. & Selinger B. Better estimates of exponential decay parameters. J. Phys. Chem. 85, 2941–2946 (1981).

[b34] Maricle D. L. & Hodgson W. G. Reducion of oxygen to superoxide anion in aprotic solvents. Anal. Chem. 37, 1562–1565 (1965).

[b35] Okoshi M., Yamada Y., Yamada A. & Nakai H. Theoretical analysis on de-solvation of lithium, sodium, and magnesium cations to organic electrolyte solvents. J. Electrochem. Soc. 160, A2160–A2165 (2013).

[b36] Ziegler M. & Madura J. Solvation of metal cations in non-aqueous liquids. J. Solution Chem. 40, 1383–1398 (2011).

[b37] Lee B. . Theoretical evidence for low charging overpotentials of superoxide discharge products in metal-oxygen batteries. Chem. Mater. 27, 8406–8413 (2015).

[b38] Petrowsky M. A. Ion Transport in Liquid Electrolytes (PhD thesis Univ. of Oklahoma (2008).

[b39] Kashchiev D. & Van Rosmalen G. Review: nucleation in solutions revisited. Cryst. Res. Technol. 38, 555–574 (2003).

[b40] Takiyama H. Supersaturation operation for quality control of crystalline particles in solution crystallization. Adv. Powder Technol. 23, 273–278 (2012).

[b41] Khetan A., Pitsch H. & Viswanathan V. Solvent degradation in nonaqueous Li-O_2_ batteries: oxidative stability versus H-abstraction. J. Phys. Chem. Lett. 5, 2419–2424 (2014). 26277809 10.1021/jz501154v

[b42] Khetan A., Luntz A. & Viswanathan V. Trade-offs in capacity and rechargeability in nonaqueous Li-O_2_ batteries: solution-driven growth versus nucleophilic stability. J. Phys. Chem. Lett. 6, 1254–1259 (2015). 26262983 10.1021/acs.jpclett.5b00324

[b43] Sawyer D. T. & Valentine J. S. How super is superoxide? Acc. Chem. Res. 14, 393–400 (1981).

[b44] Chin D. H., Chiericato G., Nanni E. J. & Sawyer D. T. Proton-induced disproportionation of superoxide ion in aprotic media. J. Am. Chem. Soc. 104, 1296–1299 (1982).

[b45] Hill G. S. . The X-ray structure of a sodium peroxide hydrate, Na_2_O_2_·8H_2_O, and its reactions with carbon dioxide: relevance to the brightening of mechanical pulps. Can. J. Chem. 75, 46–51 (1997).

[b46] Zhai D. . Raman evidence for late stage disproportionation in a Li-O_2_ battery. J. Phys. Chem. Lett. 5, 2705–2710 (2014). 26277967 10.1021/jz501323n

[b47] Zhai D. . Interfacial effects on lithium superoxide disproportionation in Li-O_2_ batteries. Nano Lett. 15, 1041–1046 (2015). 25615912 10.1021/nl503943z

[b48] Fishman M., Zhuang H. L., Mathew K., Dirschka W. & Hennig R. G. Accuracy of exchange-correlation functionals and effect of solvation on the surface energy of copper. Phys. Rev. B 87, 245402 (2013).

[b49] Mathew K., Sundararaman R., Letchworth-Weaver K., Arias T. A. & Hennig R. G. Implicit solvation model for density-functional study of nanocrystal surfaces and reaction pathways. J. Chem. Phys. 140, 084106 (2014). 24588147 10.1063/1.4865107

[b50] Burgess J. Metal Ions in Solution Chichester: Horwood Ellis (1978).

[b51] Ben-Amotz D., Raineri F. O. & Stell G. Solvation thermodynamics: theory and applications. J. Phys. Chem. B 109, 6866–6878 (2005). 16851773 10.1021/jp045090z

