# Supplementary material for: Dissolution and ionization of sodium superoxide in sodium–oxygen batteries
Source: Nat Commun. 2016 Feb 19;7:10670. doi: 10.1038/ncomms10670 (PMC4762881; doi:10.1038/ncomms10670)
Supplement: Supplementary Information — Supplementary Figures 1-11, Supplementary Notes 1-10 and Supplementary References [file ncomms10670-s1.pdf]

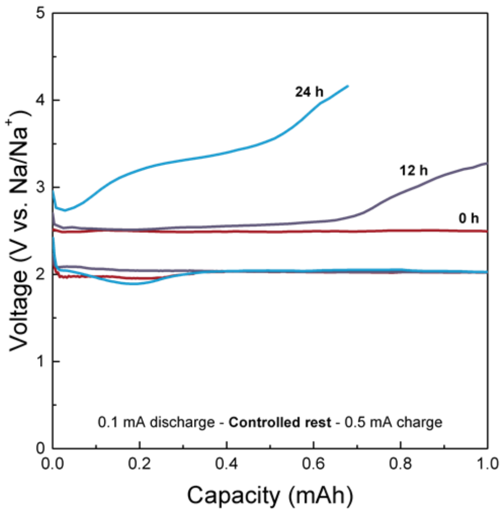

Supplementary Figure 1 | Electrochemical characteristics of Na–O_2 cells under high charge currents combined with rest time.

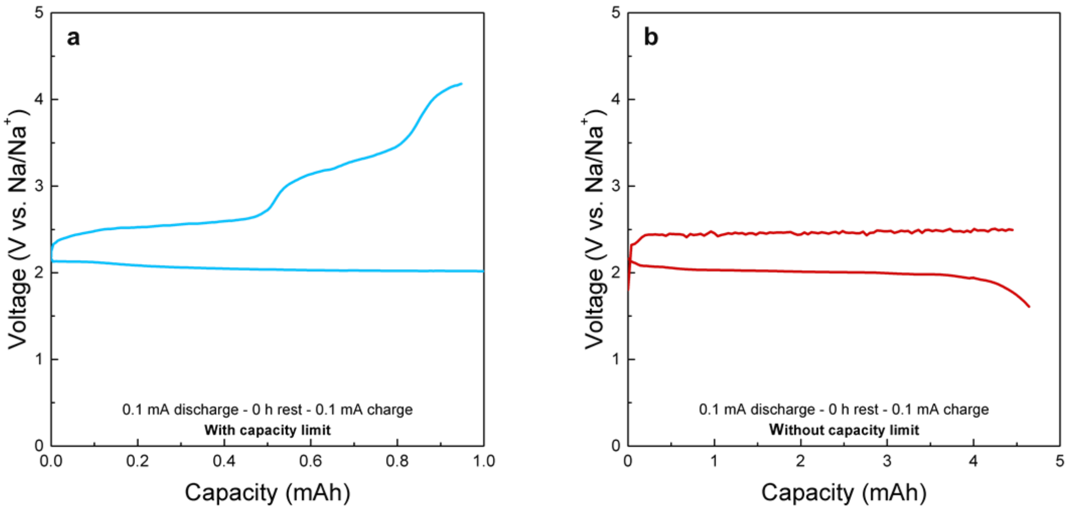

Supplementary Figure 2 | Electrochemical characteristics of Na–O_2 cells. (**a**) Operation of Na–O_2 cells with limited capacity of 1 mAh. (**b**) Full operation of Na–O_2 cells without the capacity limit. The applied currents were identical for both cells.

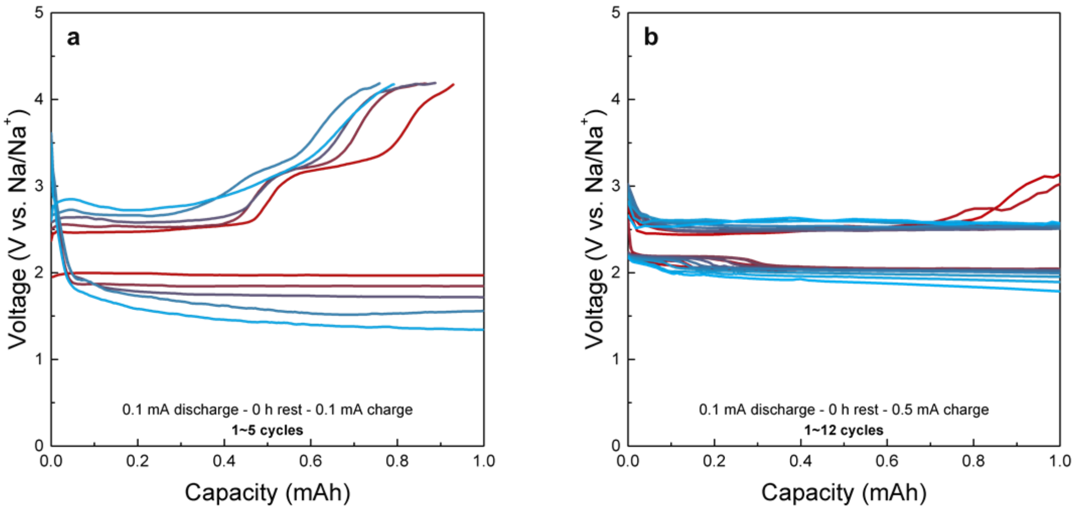

Supplementary Figure 3 | Electrochemical characteristics of Na–O_2 cells with the different voltage profiles over several cycles. (**a**) Cycling up to 5 cycles with a charge current of 0.1 mA involving clear 3-step charge profiles. (**b**) Cycling up to 12 cycles with the charge current of 0.5 mA involving the lower flat charge profiles.

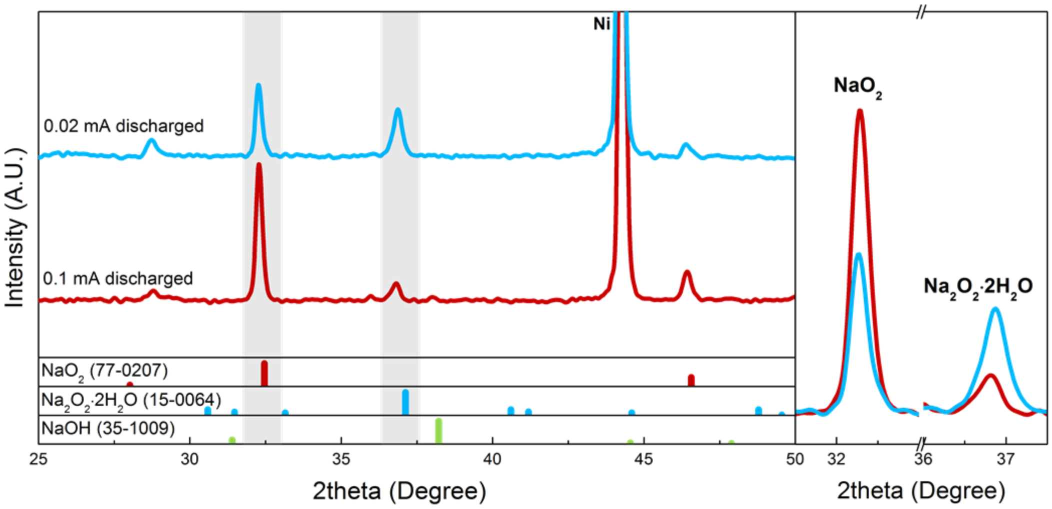

Supplementary Figure 4 | XRD spectra of the discharged cathodes of Na–O_2 cells for different discharge currents with the full discharge capacity of 4 mAh. XRD analysis clearly demonstrates that a larger amount of NaO_2 transformed into $Na_2O_2{\cdot}2H_2O$ during the discharge at the relatively smaller current of 0.02 mA because of the longer time exposure to the electrolyte.

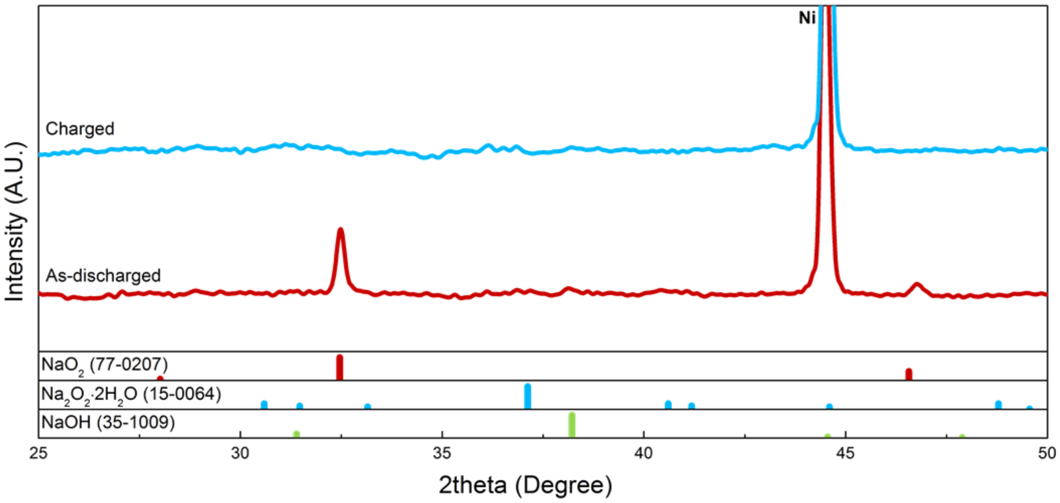

Supplementary Figure 5 | XRD analysis of the formation and decomposition of NaO_2 after the discharge and charge.

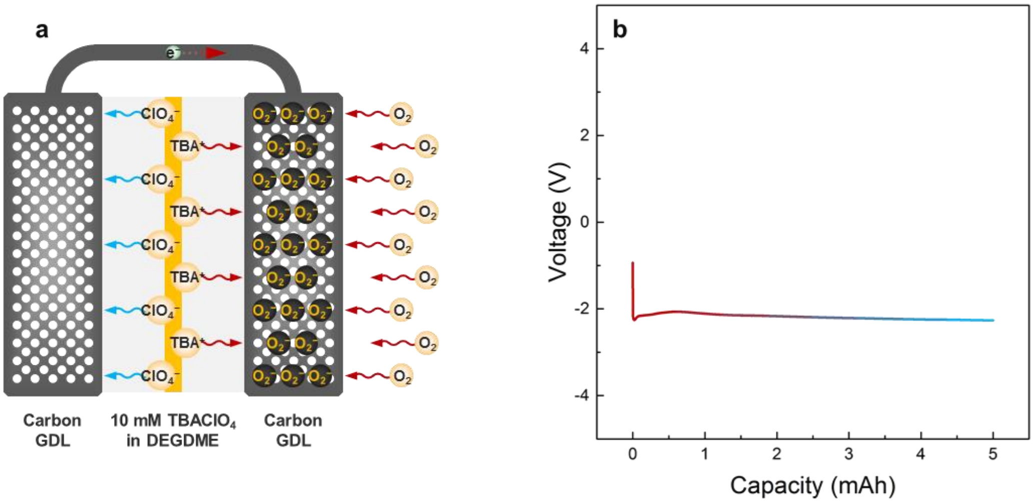

Supplementary Figure 6 | ORR experiment to simulate the generation of O_2^- *via* the dissolution and ionization of NaO_2. (**a**) Symmetric cell configuration to generate O_2^-. The electrolyte was used with 10 mM $TBAClO_4$ in DEGDME and the electrode was used with commercial carbon GDL. (**b**) The electrochemical profile of simulated ORR experiment with the symmetric cell. The applied current was 0.1 mA and the controlled capacity was 5 mAh.

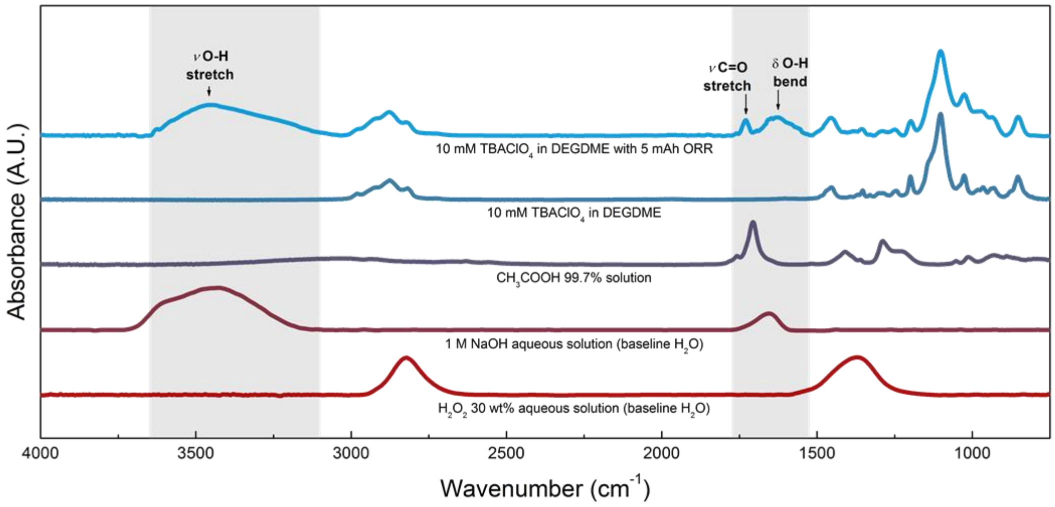

Supplementary Figure 7 | FTIR spectra of the electrolyte with simulated ORR. The measurement was carried out with ATR mode in the Ar-filled glovebox. The spectra of aqueous solutions containing H_2O_2 or NaOH as reference were measured after calibrating the baseline with pure H_2O to extract the exact bonding signals of targeting species.

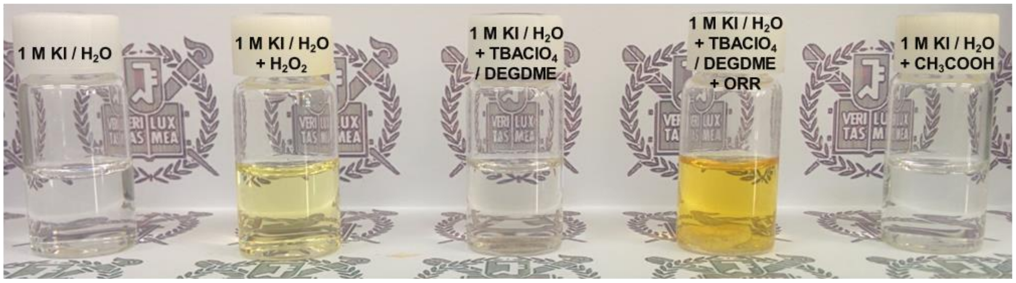

Supplementary Figure 8 | Iodometric determinations of the electrolyte with simulated ORR. The iodide/triiodide redox reaction is based on the following equation: $3\ I^{-}(aq) + 2\ H^{+}(aq) + H_2O_2(aq) \rightarrow I_3^{-}(aq) + 2\ H_2O(l)$.

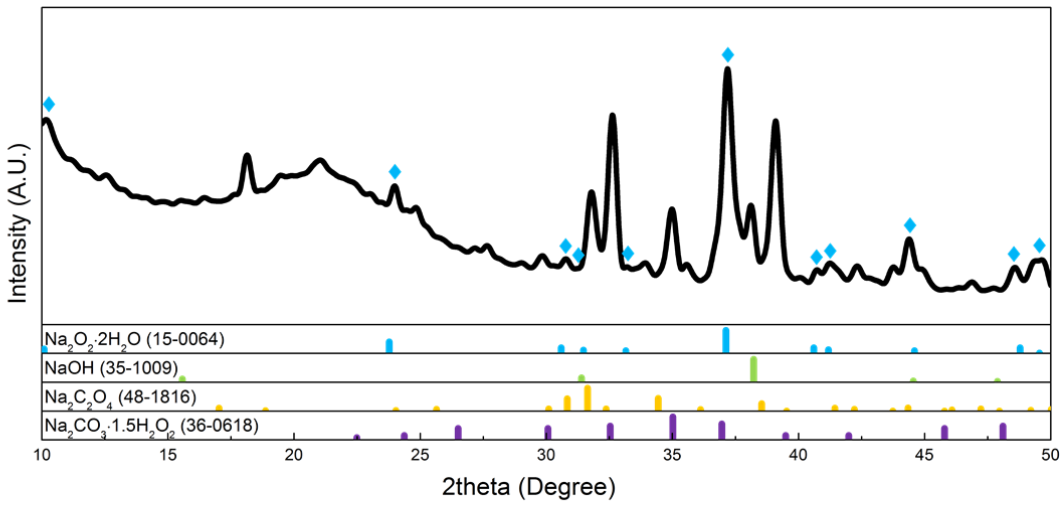

Supplementary Figure 9 | XRD pattern of the chemically synthesized $Na_2O_2 \cdot 2H_2O$ according to the proposed mechanism. All the synthesis and characterizations were carried out in the Ar-filled glovebox.

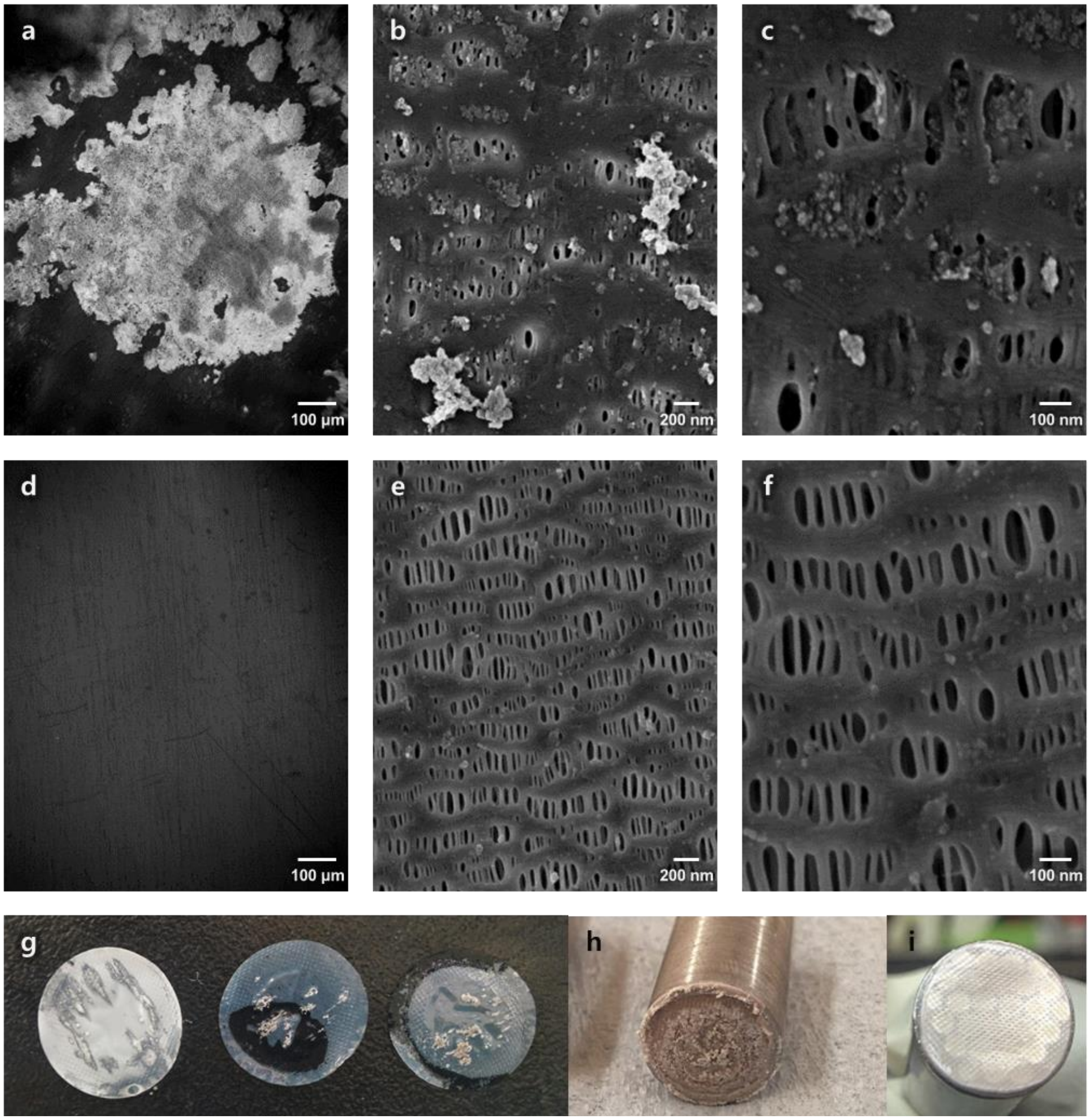

Supplementary Figure 10 | SEM images and photographs of Na dendrites after the cycling of Na–O_2 cells. (**a**–**c**) SEM images of Na dendrites after the cycling of Na–O_2 cells using direct currents; (**d**–**f**) pulse currents during the charge process. (**g**) Photographs of Na dendrites on the separators after cycling; (**h**) Na anode with the direct current. (**i**) surface of Na anode for the pulsed current.

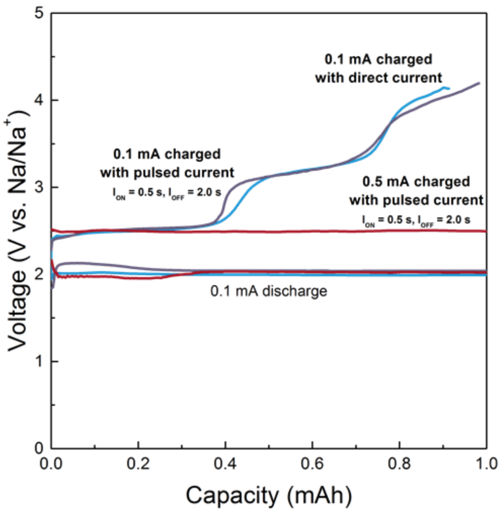

Supplementary Figure 11 | Electrochemical characteristics of Na–O_2 cells charged with direct current, pulsed current, and averaged current including the pulsed rest time.

Supplementary Note 1 (Supplementary Figure 1). The introduction of the rest time between the discharge/charge enables us to assess the effects of a non-electrochemical process on the voltage profiles, as discussed in the main discussion section. Even the high charge currents without any rest resulted in low polarization of charge; the insertion of rest time led to an increase in the charge polarizations. Thus, the effects of the chemical reactions were reconfirmed by similar electrochemical tests with relatively high charge currents. Moreover, the voltage after the 24-h rest has no flat region at 2.4 V, which is closely related to the decomposition reaction of NaO_2.

Supplementary Note 2 (Supplementary Figure 2). We also determined that the charge behaviors of Na–O_2 cells could be altered by the utilized capacities even with equally applied currents, as demonstrated in Supplementary Fig. 2. Unlike the capacity-limited cycling, the voltage profile without any capacity limit shows a low polarized charge potential over the entire capacity range, as observed in Supplementary Fig. 2b. This finding indicates that the NaO_2 formed without limited capacity was relatively stable and could be electrochemically decomposed rather than chemically deformed. Even though further analyses might be required to attain a clear understanding, we believe that the size of the discharged crystallite is also importantly affected by the dissolution of NaO_2. Thus, the longer lifetime of the large-sized NaO_2 from the fully operated discharge was attributed to the slow kinetics of dissolution in the electrolytes resulting from its low interfacial surface-to-volume ratio.

Supplementary Note 3 (Supplementary Figure 3). We also investigated the cycle properties of Na–O_2 cells depending on the shape of the charge profiles by simply controlling the charge currents. Supplementary Fig. 3 demonstrates the better reversibility achieved with the low charge potential compared with the highly polarized charge profile. The irreversible reactions with the lower charge current might be due to the chemical evolution of $Na_2O_2 \cdot 2H_2O$, which shows the 3-step charge profile.

Supplementary Note 4 (Quantitative estimation of the amount of water for the chemical transition of NaO_2). A simple reaction is expected for the phase transitions from NaO_2 to $Na_2O_2 \cdot 2H_2O$ with unauthentic H_2O:

$$2NaO_2 + 2H_2O \rightarrow Na_2O_2 \cdot 2H_2O + O_2\,(g).$$

The amount of electrochemically generated NaO_2 corresponds to the controlled capacity of 1 mAh. Thus, the calculation is

$$(1\ \text{mAh}) \times \left(3.6\ \frac{\text{C}}{\text{mAh}}\right) = \frac{3.6\ \text{C}}{96485\ \text{C mol}^{-1}} = 3.73 \times 10^{-5}\ \text{mol}$$

$$N_{NaO_2} = \left(3.73 \times 10^{-5}\ \text{mol}\right) \times \left(6.02 \times 10^{23}\ \text{mol}^{-1}\right) = 2.25 \times 10^{19}.$$

In addition, the amount of H_2O in the electrolyte used to assemble the cell (200 μL) can be estimated using the following calculation (density of electrolyte ~ 1 g/mL, H_2O content of electrolyte ~ 10 ppm):

$$(200\ \mu\text{L}) \times \left(1\ \frac{\text{g}}{\text{mL}}\right) \times (10\ \text{ppm}) = 2 \times 10^{-6}\ \text{g}_{H_2O}$$

$$N_{H_2O} = \left(\frac{2 \times 10^{-6}\ \text{g}_{H_2O}}{18\ \text{g mol}^{-1}}\right) \times \left(6.02 \times 10^{23}\ \text{mol}^{-1}\right) = 6.69 \times 10^{16}$$

$$\frac{N_{H_2O}}{N_{NaO_2}} = 0.00297.$$

The amount of H_2O in the electrolyte was only approximately 0.3% of the amount of NaO_2. Considering the given ratio of H_2O/NaO_2 and the expected reaction, the amount of H_2O is insufficient for the complete transformation of NaO_2 into $Na_2O_2 \cdot 2H_2O$. Thus, the sources of H^+ must originate from the electrolyte solvents and not the residual H_2O molecules.

Supplementary Note 5 (Calculation of pseudo-first order reaction rate from the ESR signal decay of O_2^-). In principle, the ESR reaction in Fig. 4a–c was a second-order reaction which is determined by the concentrations of two reactants such as O_2^- and the electrolyte solvent. As we investigated, the electrolyte solvent might be decomposed by nucleophilic attack of O_2^-. However, the concentrations of both reactants differentiated simultaneously, so it is rather challenging to exactly determine the reaction rate of the second-order. Nevertheless, the concentration of electrolyte solvent molecule was relatively excessive enough compared to that of O_2^-, so we could approximately ignore the concentration difference of solvent. Then, we can estimate this reaction as a pseudo-first order reaction. The reaction rate of H^+-abstraction can be expressed as

$$r = -\mathrm{d}[HO_2]/\mathrm{d}t = k[H_{(HA)}^+][O_2^-] = k'[O_2^-],\ k' = k[H_{(HA)}^+]$$

Based on the exponential fitting of the relative intensity of ESR signals, the pseudo-first order rate constant of H^+-abstraction was obtained as about $k' \approx 0.560$, and its corresponding half-life was estimated as about $t_{1/2} = \ln(2) / k' \approx 1.24$ h.

Supplementary Note 6 (Supplementary Figure 6). To experimentally simulate Reaction 4-6 in Fig. 5, firstly, we generated O_2^- in the electrolyte composed with 10 mM $TBAClO_4$

in DEGDME in the symmetric cell as shown in Supplementary Fig. 6. The simulated coulomb was 5 mAh, which is 5-fold excess amount of electrochemically formed NaO_2 with the discharge. If we exclude the shuttle effect during the discharge, the concentration of O_2^- is approximately 0.93 M compared to the volume of injected electrolyte (200 μl).

Supplementary Note 7 (Supplementary Figure 7). After allowing the relaxation of O_2^- generated in Supplementary Fig. 6 in the presence of the electrolyte, the electrolyte was examined by FTIR to identify the chemical reactions triggered by O_2^-. As compared to the as-prepared electrolyte, it was observed that the broad peak at about 3400 cm^{-1} evolves, which corresponds to νO–H stretch. This indicates the formation of free OH^- with the chemical reaction of O_2^- with the electrolyte. Furthermore, it was found that a small δO–H band and νC=O stretch at around 1625 cm^{-1} and 1728 cm^{-1}, respectively. The former signal also corresponds to the formation of the free OH^-, and the latter could be attributed to the trace amount of carboxylic functional groups (–COOH) in the byproduct. The presence of H_2O_2 was difficult to confirm from the FTIR due to the overlaps of signatures with DEGDME and will be discussed in Supplementary Fig. 8 with iodometric experiments. The identification of νO–H, δO–H, νC=O band in the electrolyte strongly support the Reaction 6 in Fig. 5.

Supplementary Note 8 (Supplementary Figure 8). We carried out the iodometric determinations to supplement the FTIR results above and verify the presence of H_2O_2 as shown in Supplementary Fig. 8. When the electrolyte exposed to O_2^- was added to the basis of 1 M KI aqueous solutions, which were initially transparent, the color of the solution was

immediately changed to yellow. It indicates the oxidation of iodide ion (I^-) to triiodide ion (I_3^-), which was induced by the presence of H_2O_2 *via* the reacttion ($2\ I^- + 2\ H^+ + H_2O_2 \rightarrow I_3^- + 2\ H_2O$). Rest of the chemical additives such as as-prepared electrolyte or CH_3COOH do not change the color of the iodide solution. This observation supports that H_2O_2 was formed after the chemical reactions coupled with ORR, and validates Reaction 7 in Fig. 5.

Supplementary Note 9 (Supplementary Figure 9). On the basis of byproducts from Reaction 6 and 7 in Fig. 5, we attempted to simulate the formation of $Na_2O_2{\cdot}2H_2O$ as proposed in Reaction 8 ($2\ NaOH + H_2O_2 \rightarrow Na_2O_2{\cdot}2H_2O$). 0.5 M H_2O_2 aqueous solution was added dropwise to the anhydrous ethanol solution of 1 M NaOH. Because NaOH is insoluble in ether-based solvents, the solvent was used with the anhydrous ethanol to reproduce the effectively dissolved state of NaOH. After the mixing, it was found that the white precipitates were immediately formed. The retrieved precipitates were examined by XRD after drying under vacuum for 30 min. Supplementary Fig. 9 identifies that the main phase of precipitates were $Na_2O_2{\cdot}2H_2O$ with a trace amount of other phases that might be formed during the process. The formation of $Na_2O_2{\cdot}2H_2O$ strongly supports the proposed Reaction 8 in Fig. 5.

Supplementary Note 10 (Supplementary Figure 10-11). In our galvanostatic cycling experiments of Na–O_2 cells, the dendritic failure of the Na metal anode was frequently observed during the charge process, similar to previous reports[1–3]. The SEM images of the separators collected after cycling with the direct currents are presented in Supplementary Figs. 10a–c, which reveal that the Na metal clogged and penetrated the pores of the

separators, resulting in the dendritic growth of the Na metal and failure of the cells. The Na dendrites were visually inspected, as shown in the photographs of the separators and anodes in Supplementary Fig. 10g. These dendrites were critically damaged during the cycling process, resulting in short circuits and potential failures. To suppress and avoid the dendritic growth of Na metal, we applied special operating protocols based on pulse-charging, which is a common methodology in electroplating and deposition[4–6]. Several researchers in the battery community have already reported on pulse-charging to suppress the dendritic growth of Li metal[7–9]. Under these conditions, the stable electrochemical cycling of Na–O_2 cells without any voltage fluctuations or sudden drops was possible. The SEM images in Supplementary Fig. 10d–f also demonstrate the absence of dendritic Na metal penetrating or clogging the pores of the separators. In addition, the upper surface of the pulse-charged Na metal in Supplementary Fig. 10i is seemingly much cleaner than that of the direct-current-charged Na metal.

Meanwhile, it was concerned that the rest time between the inter-current periods could affect our experimental observations, because one of the main key parameters for regulating the electrochemical and chemical reaction was the rest time between the discharge/charge. However, as shown in Supplementary Fig. 11, we found that the inserted resting as a pulse did not have any effect on the experimental responses. This strongly indicated that the continuously accumulated resting time during the electrochemical operation was only meaningful. The severe dendritic growth in Na metals and feasibility of pulse-charging for Na metal batteries will be discussed and reported in a separate paper.

Supplementary References

1. Bi, X. *et al.* Investigating dendrites and side reactions in sodium-oxygen batteries for improved cycle lives. *Chem. Commun.* **51**, 7665-7668 (2015).
2. Zhao, N., Li, C. & Guo, X. Long-life Na-O_2 batteries with high energy efficiency enabled by electrochemically splitting NaO_2 at a low overpotential. *Phys. Chem. Chem. Phys* **16**, 15646-15652 (2014).
3. Hartmann, P. *et al.* A comprehensive study on the cell chemistry of the sodium superoxide (NaO_2) battery. *Phys. Chem. Chem. Phys.* **15**, 11661-11672 (2013).
4. Jean-Claude Puippe, F. L. Theory and Practice of Pulse Plating. *American Electroplaters and Surface Finishers Society* (1986).
5. Chandrasekar, M. S. & Pushpavanam, M. Pulse and pulse reverse plating—Conceptual, advantages and applications. *Electrochim. Acta* **53**, 3313-3322 (2008).
6. Nielsch, K., Müller, F., Li, A. P. & Gösele, U. Uniform Nickel Deposition into Ordered Alumina Pores by Pulsed Electrodeposition. *Adv. Mater.* **12**, 582-586 (2000).
7. Yang, H., Fey, E. O., Trimm, B. D., Dimitrov, N. & Whittingham, M. S. Effects of Pulse Plating on lithium electrodeposition, morphology and cycling efficiency. *J. Power Sources* **272**, 900-908 (2014).
8. Aryanfar, A. *et al.* Dynamics of Lithium Dendrite Growth and Inhibition: Pulse Charging Experiments and Monte Carlo Calculations. *J. Phys. Chem. Lett.* **5**, 1721-1726 (2014).

9. Mayers, M. Z., Kaminski, J. W. & Miller, T. F. Suppression of Dendrite Formation via Pulse Charging in Rechargeable Lithium Metal Batteries. *J. Phys. Chem. C* **116**, 26214-26221 (2012).
